# 3-Indoleacetonitrile Is Highly Effective in Treating Influenza A Virus Infection In Vitro and In Vivo

**DOI:** 10.3390/v13081433

**Published:** 2021-07-23

**Authors:** Xuejin Zhao, Lianzhong Zhao, Ya Zhao, Kun Huang, Wenxiao Gong, Ying Yang, Li Zhao, Xiaohan Xia, Zaiyun Li, Feng Sheng, Xuezhu Du, Meilin Jin

**Affiliations:** 1 State Key Laboratory of Biocatalysis and Enzyme Engineering, School of Life Sciences, Hubei University, Wuhan 430062, China; xuejin0907@163.com (X.Z.); 18336321541@163.com (L.Z.); xxiaohan1998@163.com (X.X.); shengfsk@163.com (F.S.); 2 State Key Laboratory of Agricultural Microbiology, Huazhong Agricultural University, Wuhan 430070, China; zlz0305@webmail.hzau.edu.cn (L.Z.); zya9811@163.com (Y.Z.); kunhuang@webmail.hzau.edu.cn (K.H.); wxgong2014@aliyun.com (W.G.); f107yy@163.com (Y.Y.); 3 College of Veterinary Medicine, Huazhong Agricultural University, Wuhan 430070, China; 4 Key Laboratory of Development of Veterinary Diagnostic Products, Ministry of Agriculture, Wuhan 430070, China; 5 National Key Lab of Crop Genetic Improvement, National Center of Oil Crop Improvement (Wuhan), College of Plant Science and Technology, Huazhong Agricultural University, Wuhan 430070, China; lizaiyun@mail.hzau.edu.cn

**Keywords:** indole derivatives, 3-indoleacetonitrile, indole-3-carboxaldehyde, influenza A virus, antiviral

## Abstract

Influenza A viruses are serious zoonotic pathogens that continuously cause pandemics in several animal hosts, including birds, pigs, and humans. Indole derivatives containing an indole core framework have been extensively studied and developed to prevent and/or treat viral infection. This study evaluated the anti-influenza activity of several indole derivatives, including 3-indoleacetonitrile, indole-3-carboxaldehyde, 3-carboxyindole, and gramine, in A549 and MDCK cells. Among these compounds, 3-indoleacetonitrile exerts profound antiviral activity against a broad spectrum of influenza A viruses, as tested in A549 cells. Importantly, in a mouse model, 3-indoleacetonitrile with a non-toxic concentration of 20 mg/kg effectively reduced the mortality and weight loss, diminished lung virus titers, and alleviated lung lesions of mice lethally challenged with A/duck/Hubei/WH18/2015 H5N6 and A/Puerto Rico/8/1934 H1N1 influenza A viruses. The antiviral properties enable the potential use of 3-indoleacetonitrile for the treatment of IAV infection.

## 1. Introduction

Influenza A virus (IAV), a member of the family Orthomyxoviridae, is a highly contagious respiratory pathogen that can cause severe clinical implications in humans and many other animals [1]. Historically, human influenza has brought several pandemics worldwide and led to countless human fatalities, including 1918 H1N1, 1968 H3N2, and 2009 H1N1 [2,3,4]. Although numerous important pandemic events were reported in nearly 100 years, predicting which influenza virus subtype would breakout and when remains a significant problem. Therefore, in the future, many efforts would be required to control the spread of the influenza virus by preventing and treating this disease.

Currently, vaccination is the most effective approach to protect individuals from IAV infection [5,6]. However, vaccine efficacy is frequently jeopardized by rapid antigenic drift, necessitating yearly vaccine renewal [7]. Furthermore, side effects and ineffectiveness may limit their application [8,9,10]. Antiviral drugs, on the other hand, play important roles in the treatment and prevention of influenza. In recent decades, neuraminidase inhibitors (NAIs; oseltamivir, zanamivir, laninamivir, and peramivir) [11,12] and M2 inhibitors (rimantidine and amantadine) [13] are the recommended anti-influenza drugs in the clinic. Moreover, viral polymerase inhibitors, such as baloxavir marboxil and favipiravir, have been developed and approved for clinical use [14]. Despite this considerable improvement, the emergence of variants with drug resistance and/or reduced drug susceptibility in patients has raised broad concerns [14,15,16]. These limitations prompt us to explore novel effective antivirals with low toxicity and reduced resistance, controlling the pandemic of newly emerged and prevailing influenza viruses.

Many bioactive compounds derived from various pharmacological medicinal plants have been extensively investigated for their potentiality as viral inhibitors. For example, in East Asia, Traditional Chinese Medicine (TCM) was a good source of bioactive ingredients that have been demonstrated to have anti-influenza properties [17,18,19,20]. Strikingly, our previous studies revealed that 14-deoxy-11,12-didehydroandrographolide (DAP), a major ingredient of a TCM herb, exerted potent anti-influenza activity against multiple subtypes of influenza A virus [21,22,23]. These findings point to the potential of herb-derived compounds as novel anti-flu drugs.

Indole-based derivatives could be naturally produced from plant sources, and they are some of the most valuable heterocyclic compounds which have a broad range of biological activities, including anti-bacterial [24], anticancer [25], anti-inflammatory [26], and antiviral properties [27]. Furthermore, increasing documents have reported that indole analogs showed a potent inhibitory effect against various viral infections in recent years. For example, arbidol (also known as umifenovir), an inhibitor of virus entry and membrane fusion, is approved as a prophylactic and therapeutic agent to treat influenza and other acute respiratory viral infections in Russia and China [28,29]. Inspired by these findings, this study tested four indole derivatives for their anti-influenza activity and demonstrated 3-indoleacetonitrile as a promising drug for influenza A virus treatment.

## 2. Materials and Methods

### 2.1. Compounds

3-indoleacetonitrile (98%) (Sigma-Aldrich, 129453, St. Louis, MO, USA), indole-3-carboxaldehyde (97%) (Sigma-Aldrich, 129445), and 3-carboxyindole (99%) (Sigma-Aldrich, 284734) were purchased from Sigma-Aldrich (St. Louis, MO, USA). Arbidol hydrochloride (99.68%) (MCE, HY-14904A) and gramine (99.45%) (MCE, HY-N0166, Shanghai, China) were purchased from MedChemExpress (MCE, Shanghai, China). All compounds were dissolved in DMSO at various concentrations for specific usage.

### 2.2. Cells and Viruses

Human lung epithelial cells (A549) were maintained in F12 media (Invitrogen, Carlsbad, CA, USA) supplemented with 10% fetal bovine serum (FBS; GIBCO, Auckland, New Zealand). MDCK cells were obtained from the American Type Culture Collection (Manassas, VA, USA) and propagated in Dulbecco’s Modified Eagle’s Medium (DMEM; Invitrogen, Carlsbad, CA, USA) containing 10% FBS. All cells were cultured and maintained at 37 °C in 5% CO_2_. The virus strains A/duck/Hubei/WH18/2015 H5N6 (H5N6) and A/Hunan/Hubei/3/2005 H3N2 (H3N2) were isolated in our lab. A/Puerto Rico/8/1934 H1N1 (PR8) was conserved in our lab. A/California/04/2009 (Cal09) was provided by Wuhan Institute of Virology (Wuhan, China). Green fluorescent protein (GFP) recombinant H5N6 virus (H5N6-GFP) was constructed based on wild-type H5N6 following a previous method [30]. All viruses were amplified using 10-day-old embryonic chicken eggs. All experiments with influenza virus were carried out in a biosafety level 3 laboratory (BSL3), complying with the recommendations of the BSL3 laboratory at Huazhong Agricultural University.

### 2.3. Cell Viability Assay

CCK-8 kit was commercially obtained from the Donjindo company (Donjindo, Kumanoto, Japan) and was utilized to measure the cell viability of drug-treated A549 cells according to the provided protocol. Briefly, A549 cells were seeded into 96-well plates and cultured until the cell confluence reached 80%. Then, the cells were treated with compounds at different concentrations for 24 h. After that, CCK-8 solution was added to each well and incubated further for 2 h at 37 °C. The absorbance at 450 nm was measured using SPARK 10 M (Tecan Austria GmbH Untersbergstr, Grödig, Austria).

### 2.4. Influenza Virus Manipulation

A549 or MDCK cells were plated in a 12-well plate until the cell confluence reached 80%. Then, the cells were inoculated with indicated influenza viruses at an MOI of 0.005, followed by twice washing with PBS. One hour after inoculation, the cells were incubated with or without drugs for the indicated time. Subsequently, the supernatants’ viral titers were calculated by the determination of TCID_50_. According to the requirement, the cell pellets were subjected to flow cytometry, Western blot, or RNA extraction.

### 2.5. Flow Cytometry Analysis

Adherent A549 or MDCK cells were firstly washed with warm PBS and then digested through 0.25% trypsin with 0.02% ethylenediaminetetraacetic acid (EDTA; GENOM, Hangzhou, China). After that, the cells were dispersed thoroughly and fixed with 4% paraformaldehyde (Biosharp, Hefei, China). Finally, the GFP-positive cells were measured by flow cytometry (BD FACSVerse^TM^ Flow Cytometer, San Jose, CA, USA).

### 2.6. Real-Time Quantitative PCR (RT-qPCR) Analysis

Total RNA was isolated from cells using the TRIzol reagent (TaKaRa, Biotechnology, Dalian, China). GAPDH and viral NP mRNAs were reverse transcribed by avian myeloblastosis virus reverse transcriptase (AMV; TaKaRa) with an oligo(dT)18 primer, whereas influenza viral RNA (NP vRNA) was reverse transcribed using primer 5′-AGCAAAAGCAGG-3′. Real-time quantitative PCR (qRT-PCR) was carried out in triplicate on an ABI ViiA7 Instrument (Applied Biosystems, Foster City, CA, USA) using SYBR green detection chemistry (Roche, Basel, Switzerland). The amplification conditions were: 95 °C for 30 s, followed by 45 cycles of 95 °C for 5 s, 60 °C for 30 s. Relative expression levels were calculated by applying the 2^−ΔCt^ method using GAPDH as the reference gene. Primers used in real-time qPCR were as follows: GAPDH, forward: 5′-GGAGCGAGATCCCTCCAAAAT-3′, reverse: 5′-GGCTGTTGTCATACTTCTCATGG-3′; NP, forward: 5′-AACGACCGGAATTTCTGGAGAGG-3′, reverse: 5′-CCGTACACACAAGCAGGCAAGC-3′.

### 2.7. Western Blotting Analysis

The Western blot procedures that were conducted complied with the protocol described in our previous study [31]. The antibodies specific for probing influenza NP and PB2 proteins were provided by GeneTex (Irvine, CA, USA), anti-GAPDH antibody (#CB100127) was purchased from California Biosciences (Cali-Bio, Coachella, CA, USA), which was used as a loading control for protein. The relative integrated optical density (RIOD) of all blots was determined using Image-Pro Plus software (Media Cybernetics, Rockville, MD, USA); the value was provided below each blot.

### 2.8. Mouse Experiments

Eight-week-old SPF BALB/c female mice were purchased from the Animal Experimental Centre of Huazhong Agricultural University (Wuhan, China). All animal experiments were approved by the Hubei Provincial Animal Care and Use Committee (approval numbers: SYXK 2015-0084, approval date: 21 October 2015).

Before the virus challenge, the toxicity of 3-indoleacetonitrile to mice was evaluated. Eight-week-old SPF BALB/c female mice (*n* = 3 per group) were individually administered with various concentrations of 3-indoleacetonitrile or DMSO (vehicle control) for 10 consecutive days. The physical appearance of each mouse was observed daily, and the body weights and survival of mice were also recorded. To determine the effect of 3-indoleacetonitrile on mice’s viral infection mortality, 3 groups of 8-week-old female BALB/c mice were treated with 3-indoleacetonitrile (20 mg/kg) or DMSO (0.5%) via tail-vein injection. Two hours later, the mice were intranasally inoculated with or without 50 μL of 2 median lethal doses (LD_50_) of H5N6 or PR8 viruses (designated as Day 0). After that, the mice were administered with 20 mg/kg 3-indoleacetonitrile or DMSO on day 1/3/5/7/9 post-infection. The body weights and survival of the mice in each group were monitored daily for 14 days post-infection (dpi). Another 3 groups of mice were independently set for sample preparation, and three mice per group were euthanized at 7 dpi. The right lungs of the mice were removed to determine the virus titers, and the left lungs were fixed in formalin for histopathological analysis. 

### 2.9. Statistics

Data were shown as means ± SD collected from three independent experiments. The significance value was calculated using the Student’s *t*-test or two-way ANOVA. *p* < 0.05 was considered statistically significant.

## 3. Results

### 3.1. Screening 3-Indoleacetonitrile as an Antiviral Agent against IAV Infection

**Figure 1:**
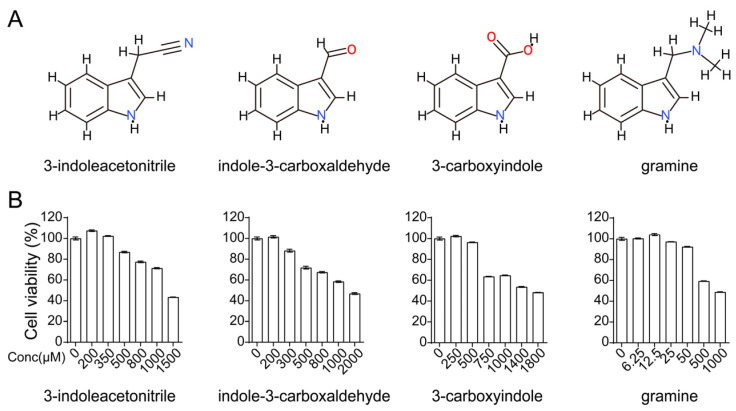
Cytotoxicity of indole derivatives in A549 cells. (**A**) Molecular structure of 3-indoleacetonitrile, indole-3-carboxaldehyde, 3-carboxyindole, and gramine. (**B**) A549 cells were treated with 3-indoleacetonitrile, indole-3-carboxaldehyde, 3-carboxyindole, or gramine using the indicated concentrations for 24 h. Then, the cell viability was measured by CCK-8.

Indole derivatives have been shown to restrict viral replication effectively in vitro and in vivo, which motivated us to find new indole compounds as novel potential anti-IAV agents. 3-indoleacetonitrile, indole-3-carboxaldehyde, and 3-carboxyindole are the chemical constituents of Banlangen, a well-known TCM for its broad antiviral activities [32,33]. Furthermore, gramine was also included in the investigation for its similar structure (Figure 1A). Accordingly, these four indole derivatives were subjected to antiviral screening using a GFP-fused influenza A virus (H5N6-GFP). Before testing, we evaluated the cytotoxicity of these four compounds in A549 cells and determined the best concentration for further evaluation (Figure 1B). The maximum concentrations without cytotoxicity were used for follow-up evaluation, which may achieve the best antiviral effect. 

**Figure 2:**
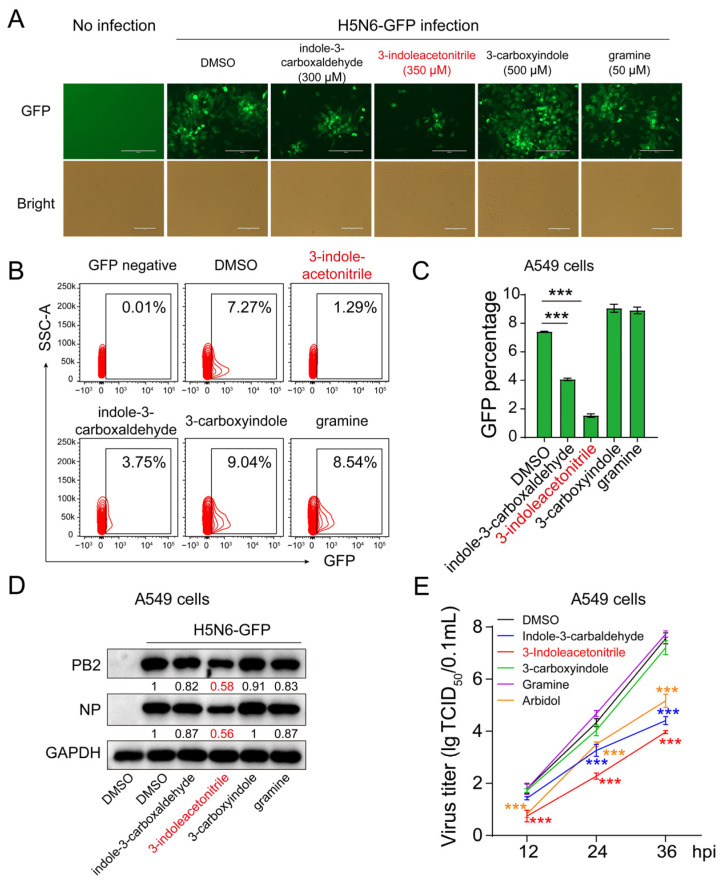
The effect of indole derivatives on H5N6-GFP virus replication. The A549 cells were infected with the H5N6-GFP virus at an MOI of 0.005, followed by treatment with 3-indoleacetonitrile, indole-3-carboxaldehyde, 3-carboxyindole, and gramine at indicated concentrations for 24 h. After that, the GFP intensity was acquired using fluorescence microscopy (**A**). The percentage of GFP-positive cells was calculated through flow cytometry (**B**, a presentative image) (**C**, data collected from three independent biological experiments). The viral PB2 and NP proteins were analyzed by Western blotting (**D**). The growth curves of H5N6-GFP virus in supernatants were determined based on three time points of infection as indicated (**E**), 3-indoleacetonitrile (350 μM); indole-3-carboxaldehyde (300 μM); 3-carboxyindole (500 μM); gramine (50 μM); arbidol (8 μM). *** *p* < 0.001; calculated from three independent experiments by two-tailed Student’s *t*-test (**C**) or two-way ANOVA (**E**).

Next, we infected A549 cells with H5N6-GFP virus at an MOI of 0.005 followed by incubation with four compounds or DMSO individually for 24 h. Under microscopy, H5N6-GFP caused no visible cytopathic effect in all infected cells compared to non-infected cells (Figure 2A). However, the densities of GFP fluorescence were significantly decreased upon treatment with 3-indoleacetonitrile or indole-3-carboxaldehyde, but not 3-carboxyindole, or gramine compounds, by comparing with the DMSO control (Figure 2A). We further measured the population of GFP-positive cells using flow cytometry, showing an inhibited viral proliferation in 3-indoleacetonitrile or indole-3-carboxaldehyde-treated cells (Figure 2B,C), in line with the observation under microscopy. Moreover, the effect of these compounds on virus replication was further confirmed by measuring the cellular viral PB2 and NP protein levels (Figure 2D).

Arbidol is an approved antiviral drug for clinical use, containing an indole backbone (Figure S1A). We next intended to compare the antiviral activity of 3-indoleacetonitrile or indole-3-carboxaldehyde with that of arbidol. In A549 cells, we used 8 μM of arbidol for subsequent assays in that 12 μM of arbidol treatment showed severe cytotoxicity under microscopy (data not shown), which was also indicated by CCK8 assay (Figure S1B). Accordingly, we titered the viral load in the culture supernatant of H5N6-GFP-infected cells incubated with indicated compounds, demonstrating a similar antiviral activity between indole-3-carboxaldehyde and arbidol under a non-toxic dose (Figure 2E). Strikingly, 3-indoleacetonitrile presented more enhanced antiviral activity than both indole-3-carboxaldehyde and arbidol. In addition, 3-indoleacetonitrile caused a comparable reduction in PR8 PB2 and NP proteins compared to arbidol (Figure S1C).

**Figure 3:**
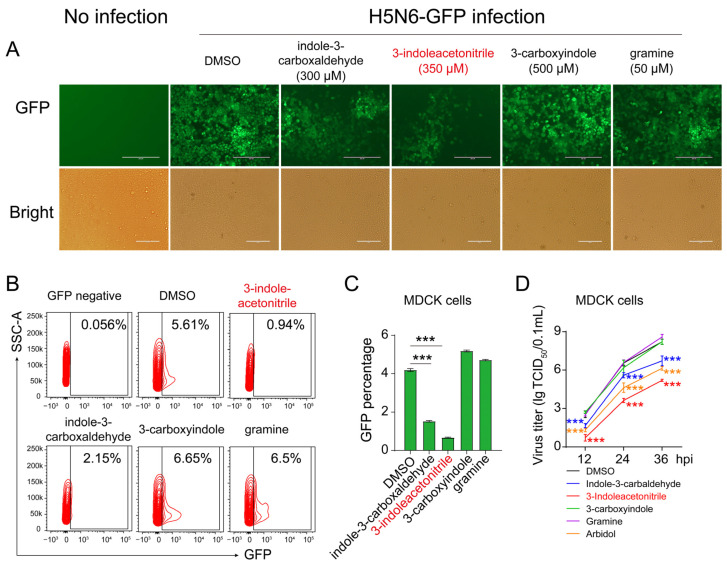
The effect of indole derivatives on H5N6-GFP virus replication in MDCK cells. The MDCK cells were infected with the H5N6-GFP virus at an MOI of 0.005, followed by treatment with 3-indoleacetonitrile, indole-3-carboxaldehyde, 3-carboxyindole, and gramine at indicated concentrations for 24 h. After that, the GFP intensity was acquired using fluorescence microscopy (**A**). The percentage of GFP-positive cells was calculated through flow cytometry (**B**, a presentative image) (**C**, data collected from three independent biological experiments). The growth curves were determined by measuring H5N6-GFP virus yield in supernatants at time points of 12, 24, and 36 hpi (**D**), 3-indoleacetonitrile (350 μM); indole-3-carboxaldehyde (300 μM); 3-carboxyindole (500 μM); gramine (50 μM); arbidol (8 μM). *** *p* < 0.001; calculated from three independent experiments by two-tailed Student’s *t*-test (**C**) or two-way ANOVA (**D**).

Additionally, to further validate our observations, we carried out similar experiments in MDCK cells using the same procedures. In line with the observations on A549 cells, 3-indoleacetonitrile also showed more potent anti-influenza activity than indole-3-carboxaldehyde in MDCK cells; however, 3-carboxyindole and gramine did not affect viral replication (Figure 3A–C). Notably, under a non-toxic dose, 3-indoleacetonitrile exhibited more favorable antiviral activity than arbidol (Figure 3D). Together, these results suggested that 3-indoleacetonitrile and indole-3-carboxaldehyde hold potential as anti-influenza entities. Here, 3-indoleacetonitrile was subjected to a follow-up evaluation due to its more potent inhibitory effect on virus replication.

### 3.2. 3-Indoleacetonitrile Exerts Broad-Spectrum Activity against the Influenza A Virus

**Figure 4:**
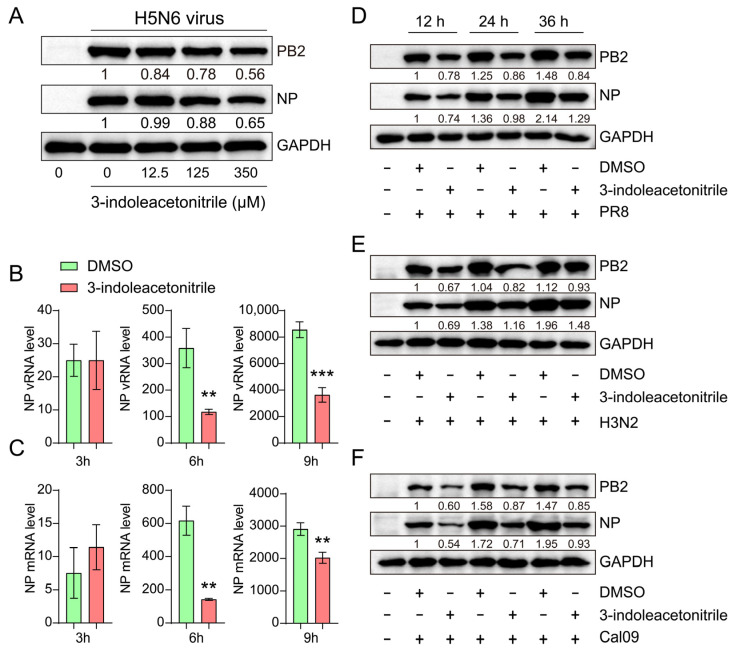
3-indoleacetonitrile inhibits influenza virus replication with a broad spectrum. (**A**) The protein level of H5N6 NP and PB2 was measured in A549 cells treated with an increased dose of 3-indoleacetonitrile. (**B**,**C**) The effect of 3-indoleacetonitrile on H5N6 vRNA (**B**) and mRNA (**C**) abundance at 3, 6, and 9 hpi. (**D**–**F**) Effect of 3-indoleacetonitrile on viral PB2 and NP protein level in A549 cells infected with PR8 (**D**), H3N2 (**E**), or Cal09 (**F**) viruses. ** *p* < 0.01; *** *p* < 0.001; calculated from three independent experiments by two-tailed Student’s *t*-test.

Based on the above results, we asked whether 3-indoleacetonitrile could restrict the replication of wild-type H5N6 virus and other subtypes of influenza virus. As shown in Figure 4A, in H5N6 virus-infected cells, the viral NP and PB2 proteins decreased gradually with the increased dose of 3-indoleacetonitrile. We also assessed the impact of 3-indoleacetonitrile on viral RNA synthesis in the early stage of virus infection. At 3 h post-infection (hpi), 3-indoleacetonitrile caused no difference in the relative amount of NP vRNA and mRNA. However, at 6 and 9 hpi, NP vRNA and mRNA yield was profoundly reduced in the presence of 3-indoleacetonitrile (Figure 4B,C). This result suggested that 3-indoleacetonitrile started its function in the initial stage of infection. Next, we selected PR8 H1N1, H3N2, and California09 H1N1 strains to convince our conclusion. We demonstrated that 3-indoleacetonitrile impaired these three tested viruses’ PB2 and NP synthesis at 12, 24, and 36 hpi (Figure 4D–F). Collectively, 3-indoleacetonitrile exhibits broad-spectrum antiviral activity against IAV infection.

### 3.3. 3-Indoleacetonitrile Reduces Viral Replication in Mice

**Figure 5:**
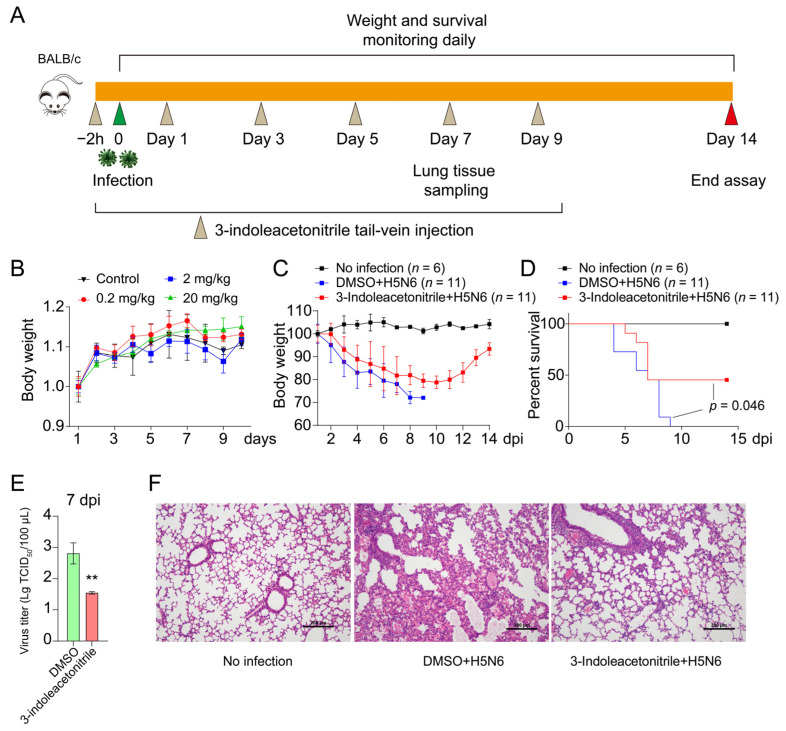
3-indoleacetonitrile restricts H5N6 virus replication in mice. (**A**) Schematic diagram of the mouse experiment. (**B**) Female BALB/c mice were treated with 3-indoleacetonitrile (0.2, 2 or 20 mg/kg per day) or 0.5% DMSO via tail-vein injection. Body weights in each group were monitored daily for 10 days post-treatment. (**C**–**F**) Female BALB/c mice were inoculated with 50 μL of 2 LD_50_ H5N6 viruses and treated with 20 mg/kg 3-indoleacetonitrile or 0.5% DMSO. In the next 2 weeks, the body weights (**C**) and mouse survival (**D**) were recorded daily. *p*-value was calculated using the log-rank (Mantel–Cox) test. On 7 dpi, the left lung’s viral titers (**E**) were measured by determining the TCID_50,_ and the right lungs were fixed for H&E staining (**F**). ** *p* < 0.01; calculated from three independent experiments by two-tailed Student’s *t*-test.

To evaluate the anti-influenza activity of 3-indoleacetonitrile in vivo, we firstly tested the toxicity of 3-indoleacetonitrile on mice. We treated the mice with DMSO or 3-indoleacetonitrile at concentrations of 0.2 mg/kg, 2 mg/kg, or 20 mg/kg per mouse for 10 days. In this period, mouse body weights and health conditions were monitored daily, showing no difference between 3-indoleacetonitrile-treated groups and the control group (Figure 5B). As expected, exposure to 3-indoleacetonitrile did not cause distinguishable pathological changes in the mouse organs, including lung, liver, spleen, and kidney (Figure S2). These observations suggested that the concentration as high as 20 mg/kg seemed to be non-toxic to mice and thus was used for the following experiments. Subsequently, as shown in Figure 5A, we inoculated the mice with H5N6 or PR8 viruses at a dose of 2 LD_50_. Two hours later, mice were injected with 3-indoleacetonitrile or DMSO at a concentration of 20 mg/kg via tail-vein, followed by additional injection on 1/3/5/7/9 dpi. In the next 14 consecutive days, we observed that 3-indoleacetonitrile-treated mice exhibit less weight loss than DMSO-treated mice (Figure 5C and Figure S3A). More importantly, H5N6 and PR8-infected mice treated with the vehicle all died before 9 dpi. By contrast, 3-indoleacetonitrile intake increased the survival rate of H5N6-infected mice to 45.45% (5 out of 11 mice survived) and PR8-infected mice to 50% (four out of eight mice survived) (Figure 5D and Figure S3B). Consistent with these results, the viral load in the lungs of 3-indoleacetonitrile-treated mice was significantly reduced compared to DMSO-treated mice (Figure 5E and Figure S3C). In addition, 3-indoleacetonitrile reduced the lung lesions caused by viral infection (Figure 5F and Figure S3D). Taken together, 3-indoleacetonitrile could restrict influenza replication in mice.

## 4. Discussion

Multiple subtypes of IAV give rise to the ineffectiveness of specific influenza vaccines, and long-term antiviral exposure can readily develop drug resistance, motivating us to find new antiviral remedies. Compounds generated in nature have shown great promise in treating this disease because of their potential inhibitory properties and relatively low cytotoxicity [17,18,19,20]. For example, our previous studies have demonstrated that 14-deoxy-11,12-didehydroandrographolide, an ingredient of *Andrographis paniculate*, impeded various subtypes of influenza viruses’ replication by inhibiting apoptosis and excessive inflammatory responses [21,22,23]. Accumulating studies have demonstrated that indole derivatives exhibit great potential to restrict IAV infection. Indole derivatives could be synthesized from tryptophan in plants. We chose 3-indoleacetonitrile, indole-3-carboxaldehyde, and 3-carboxyindole for evaluation since these three indole derivatives could be produced by Cruciferous (or Brassica) vegetables, whose roots and leaves were proved to contain antiviral constituents [32,33,34]. Gramine, produced by many higher plants such as giant reed [35], barley [36], and reed canarygrass [37], was another candidate for its similar structure with the above three compounds. Notably, 3-indoleacetonitrile showed the strongest inhibitory effect on virus replication in cultured cells and mice among these tested compounds.

Before the examination of virus replication, we tested the cytotoxicity of these four compounds in A549 cells. A higher but non-toxic concentration was applied to all the compounds. Subsequently, using H5N6-GFP as a tool virus, we demonstrated that both 3-indoleacetonitrile and indole-3-carboxaldehyde reduced GFP fluorescence based on microscopy and flow cytometry results. Consistently, viral PB2 and NP proteins were also declined by 3-indoleacetonitrile and indole-3-carboxaldehyde in infected A549 cells. Interestingly, 3-indoleacetonitrile appeared to exhibit more potent anti-influenza activity than indole-3-carboxaldehyde. This phenotype could be reproduced in the MDCK cell line, suggesting the antiviral activity of these two compounds without cell specificity. Notably, 3-carboxyindole, aldehyde replacement with carboxyl in indole-3-carboxaldehyde, has no inhibitory effect on viral replication. Gramine displayed no antiviral activity against IAV infection, and Wei et al. previously indicated that no inhibitory effect of gramine was observed on enterovirus 71 virus replication [38]. Together, our results indicate that 3-indoleacetonitrile and indole-3-carboxaldehyde hold great potency to restrict influenza replication. Interestingly, a previous study reported that 3-indoleacetonitrile and indole-3-carboxaldehyde decreased the biofilm formation of both *E. coli* O157:H7 and *P. aeruginosa* [39]. Our data and the others’ suggested that these two indole derivatives have multiple biological activities in pathogen defense.

To further confirm the anti-influenza activity of 3-indoleacetonitrile, we conducted additional experiments using wild-type H5N6, PR8 H1N1, H3N2, and California09 H1N1 influenza viruses. We demonstrated that 3-indoleacetonitrile impeded all these viruses’ replication in A549 cells by measuring PB2 and NP protein expression. Considering the genetic discrepancies between the tested viruses, we speculated that 3-indoleacetonitrile might utilize a common mechanism to restrict their proliferation. A previous report by S. Lee et al. suggested that indole derivative-mediated pro-inflammatory cytokine release has contributed to the inhibition of hepatitis C virus replication [40]. However, we did not observe a promoted cytokine or immune response in A549 cells upon 3-indoleacetonitrile or indole-3-carboxaldehyde treatment by measuring cellular RIG-I, IFN-β, and ISG mRNA levels (data not shown). These results exclude the possibility that 3-indoleacetonitrile prevents influenza virus replication through immune enhancement. Importantly, our q-PCR data demonstrated that the viral RNA synthesis was declined upon 3-indoleacetonitrile treatment, at least earlier than 6 hpi. One possibility is that 3-indoleacetonitrile would have compromised the viral polymerase activity in the early stage of the viral life cycle. However, this hypothesis needs to be further confirmed experimentally. 

Similarly, other indole derivatives were synthesized and investigated to restrain the replication of various viruses, including influenza [41], dengue [42], enterovirus 71 [38], and Hepatitis B virus [43]. Importantly, we noted that arbidol, an indole derivative, was an outstanding representative for influenza treatment [27] and thus was set as a positive control in this study. Based on the cell viability assay results, the 50% cytotoxic concentration of arbidol was between 48 and 96 μM, much lower than 1000 to 1500 μM of 3-indoleacetonitrile. This result suggests that 3-indoleacetonitrile has less cytotoxicity than arbidol. Furthermore, our results demonstrated that 3-indoleacetonitrile (350 μM) elicited more potent antiviral activity than arbidol (8 μM) using a dose without cytotoxicity. Although 3-indoleacetonitrile exhibited a less effective antiviral effect, a cytotoxic concentration (higher than 8 μM) was needed for arbidol to achieve a similar antiviral activity to the former. In this study, few efforts were made regarding the mechanism for the action of 3-indoleacetonitrile on the influenza virus. Previous studies illustrated that arbidol could associate with viral HA and result in an arbidol–HA complex, thereby inhibiting influenza-mediated membrane fusion [28,29]. These findings provided valuable clues regarding the mechanisms for the action of 3-indoleacetonitrile on IAV infection. 

Lastly, in line with the in vitro results, we also observed the protective role of 3-indoleacetonitrile against the infection of various influenza viruses (such as H5N6 and PR8 H1N1) in a mouse model, as determined by measuring body weight loss, survival rate, lung lesions, and viral load. These results indicated the favorable bioactivity and non-cytotoxicity of 3-indoleacetonitrile in vivo. Notably, in this study, the mice were administered with 3-indoleacetonitrile via tail-vein injection. Whether it is effective when administered through an alternative way remains to be further evaluated. Together, these findings suggest that indole moiety-based compounds, especially natural products, provide a great opportunity and possibility for developing new remedies that are effective against virus infection.

Overall, we demonstrated that 3-indoleacetonitrile exhibited potent inhibitory effects against influenza infection both in vitro and in vivo, suggesting its broad prospects in controlling the spread of the influenza virus. Despite these preliminary findings, however, the exact mechanism responsible for the effects of 3-indoleacetonitrile during viral infection warrants our additional investigation.

## Data Availability

Not applicable.

## Supplementary Materials

The following are available online at https://www.mdpi.com/article/10.3390/v13081433/s1, Figure S1: Antiviral activity of 3-indoleacetonitrile versus arbidol against PR8 virus, Figure S2: Representative images of H&E staining of the mouse tissues treated with vehicle control or various concentrations of 3-indoleacetonitrile, Figure S3: 3-indoleacetonitrile restricts PR8 virus replication in mice.
