# Supplementary material for: 3-Indoleacetonitrile Is Highly Effective in Treating Influenza A Virus Infection In Vitro and In Vivo
Source: Viruses. 2021 Jul 23;13(8):1433. doi: 10.3390/v13081433 (PMC8402863; doi:10.3390/v13081433)

3-Indoleacetonitrile Is Highly Effective In Treating Influenza A Virus Infection In Vitro and In Vivo

Xuejin Zhao [1,†], Lianzhong Zhao [2,3,4,†], Ya Zhao [2,3,4], Kun Huang [2,3,4], Wenxiao Gong [2,3,4], Ying Yang [2,3,4], Li Zhao [1], Xiaohan Xia [1], Zaiyun Li [5], Feng Sheng [1], Xuezhu Du [1,*] and Meilin Jin [2,3,4,*]

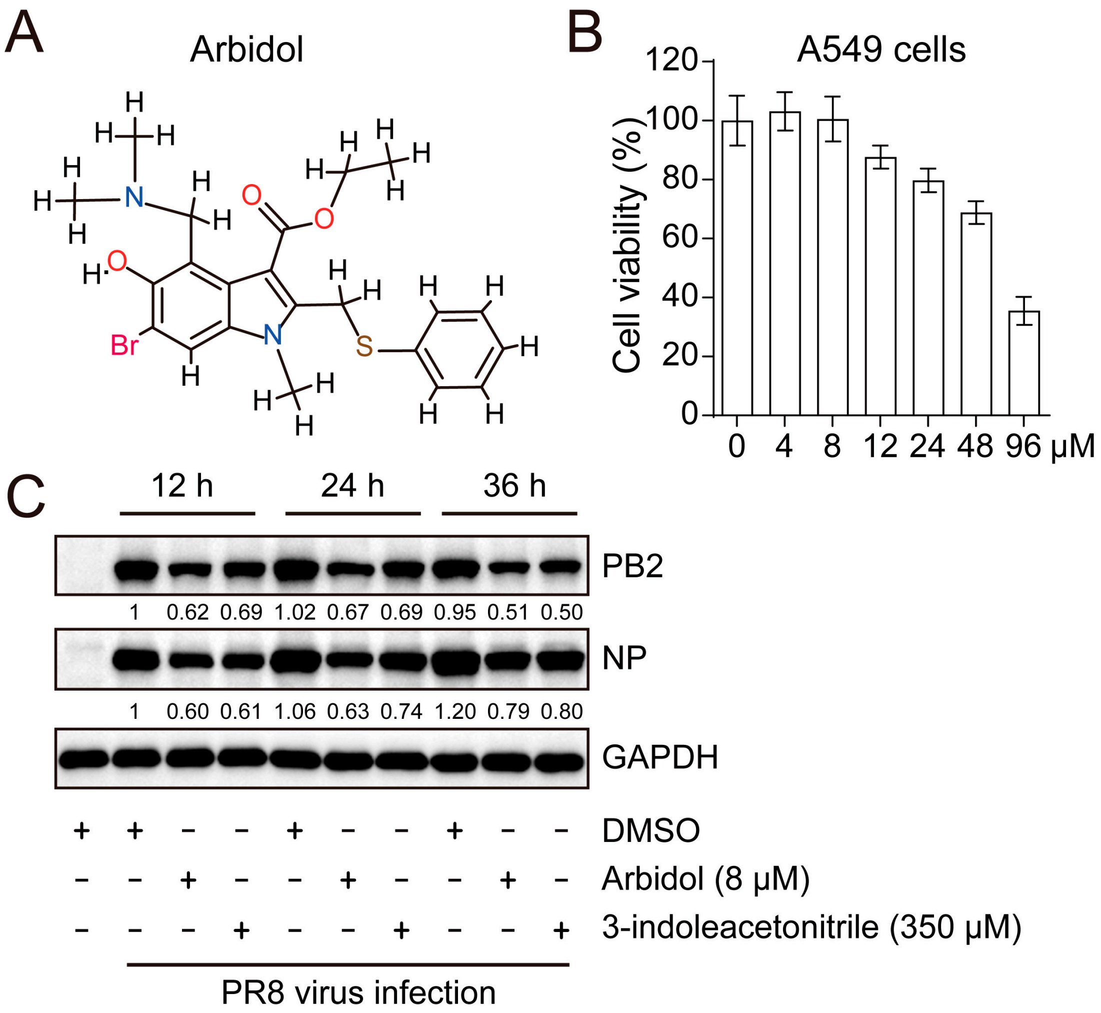

Figure S1. Antiviral activity of 3-indoleacetonitrile versus arbidol against PR8 virus. (**A**) Molecule structure of arbidol. (**B**) The A549 cells were treated with arbidol at indicated concentrations for 24 hours. Then, the cell viability was measured by CCK-8 assay. (**C**) The protein levels of PR8 viral NP and PB2 were measured in infected A549 cells treated with arbidol (8 μM) or 3-indoleacetonitrile (350 μM) at time points of 12, 24, and 36 hpi, DMSO was used as control.

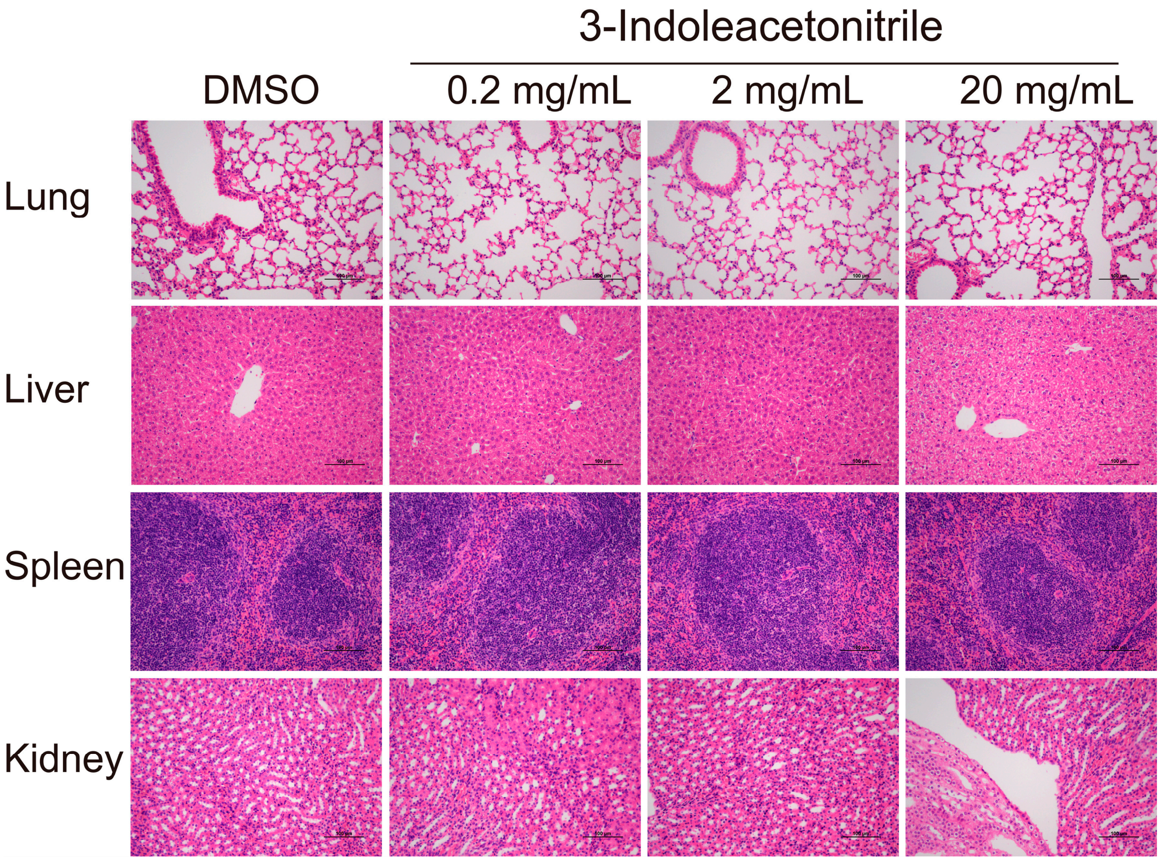

Figure S2. Representative images of H&E staining of the mouse tissues treated with vehicle control or various concentrations of 3-indoleacetonitrile. The scale bar represents 100 μm.

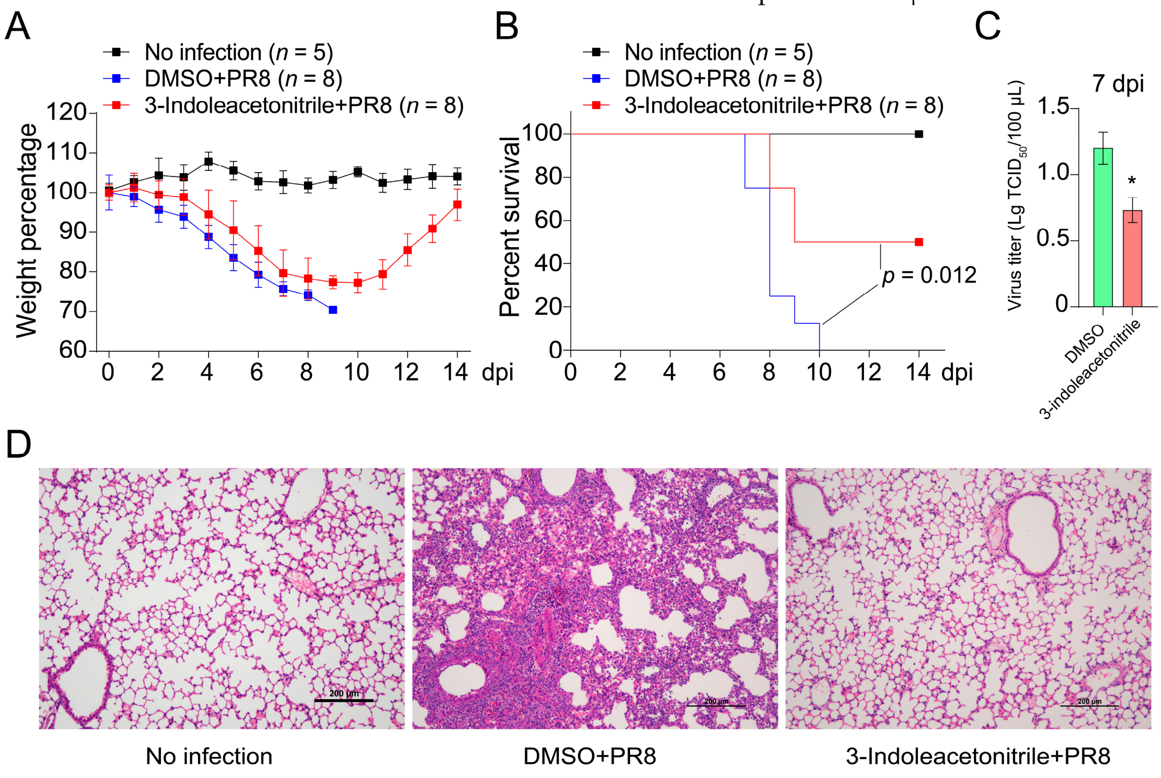

Figure S3. 3-indoleacetonitrile restricts PR8 virus replication in mice. Female BALB/c mice were inoculated with 50 μL of 2 LD_{50} PR8 viruses and treated with 3-Indoleacetonitrile or DMSO at a concentration of 20 mg/kg as indicated in Figure 5A. In the next 2 weeks, the body weights (**A**) and mouse survival (**B**) were recorded daily. *P*-value was calculated using the log-rank (Mantel-Cox) test. On 7 dpi, the left lung's viral titers (C) were measured by determining the $TCID_{50}$, and the

right lungs were fixed for H&E staining (**D**), the scale bar represents 200 μm. * $P < 0.05$; calculated from three independent experiments by two-tailed Student's t-test.

Original images of Western blotting

Figure 2D

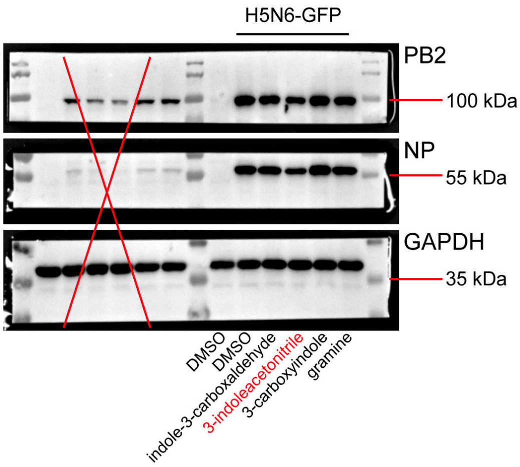

Figure 3A

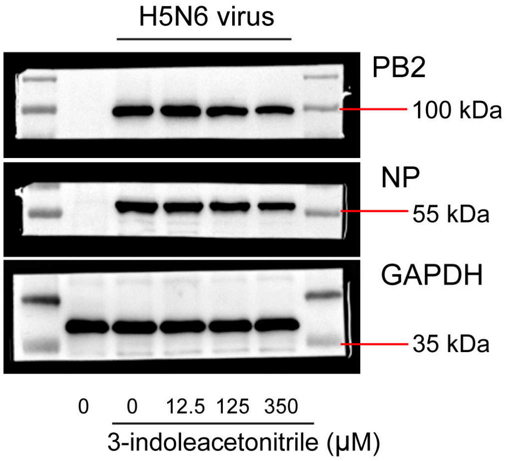

Figure 3D

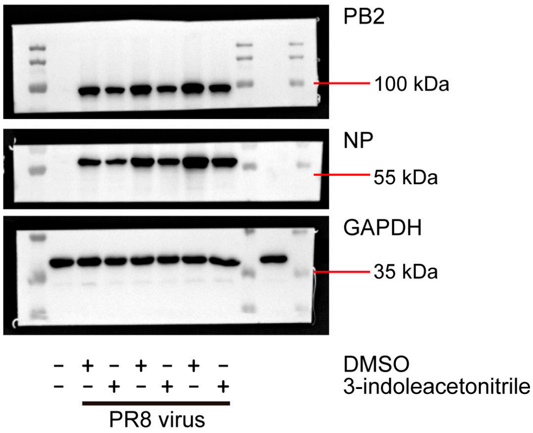

Figure 3E

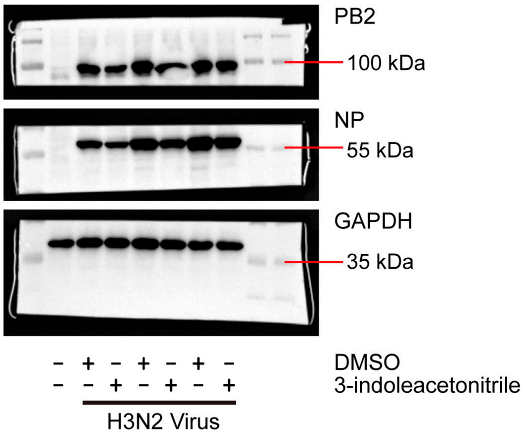

Figure 3F

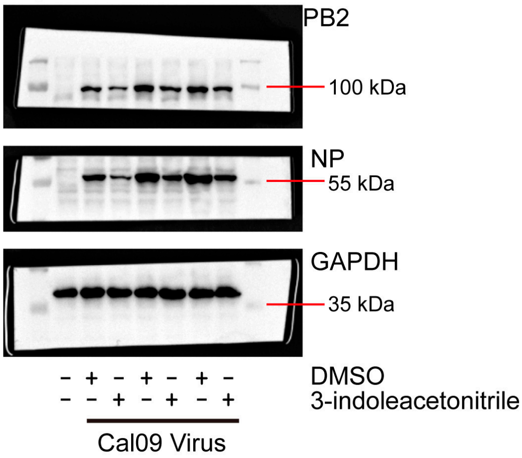

Supplementary Figure 1C

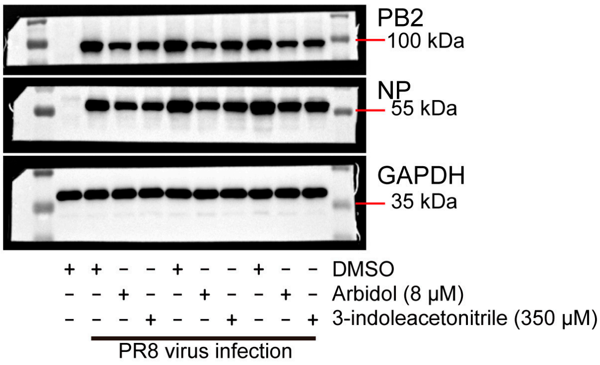

Supplement: Supplementary file 1 [file viruses-13-01433-s001.zip › viruses-1220182-supplementary.pdf]
